# Using species distribution models to predict potential hot-spots for Rift Valley Fever establishment in the United Kingdom

**DOI:** 10.1371/journal.pone.0225250

**Published:** 2019-12-23

**Authors:** Robin R. L. Simons, Simon Croft, Eleanor Rees, Oliver Tearne, Mark E. Arnold, Nicholas Johnson

**Affiliations:** 1 Animal and Plant Health Agency, New Haw, Surrey, United Kingdom; 2 National Wildlife Management Centre, Animal and Plant Health Agency, Sand Hutton York, United Kingdom; Faculty of Science, Ain Shams University (ASU), EGYPT

## Abstract

Vector borne diseases are a continuing global threat to both human and animal health. The ability of vectors such as mosquitos to cover large distances and cross country borders undetected provide an ever-present threat of pathogen spread. Many diseases can infect multiple vector species, such that even if the climate is not hospitable for an invasive species, indigenous species may be susceptible and capable of transmission such that one incursion event could lead to disease establishment in these species. Here we present a consensus modelling methodology to estimate the habitat suitability for presence of mosquito species in the UK deemed competent for Rift Valley fever virus (RVF) and demonstrate its application in an assessment of the relative risk of establishment of RVF virus in the UK livestock population. The consensus model utilises observed UK mosquito surveillance data, along with climatic and geographic prediction variables, to inform six independent species distribution models; the results of which are combined to produce a single prediction map. As a livestock host is needed to transmit RVF, we then combine the consensus model output with existing maps of sheep and cattle density to predict the areas of the UK where disease is most likely to establish in local mosquito populations. The model results suggest areas of high suitability for RVF competent mosquito species across the length and breadth of the UK. Notable areas of high suitability were the South West of England and coastal areas of Wales, the latter of which was subsequently predicted to be at higher risk for establishment of RVF due to higher livestock densities. This study demonstrates the applicability of outputs of species distribution models to help predict hot-spots for risk of disease establishment. While there is still uncertainty associated with the outputs we believe that the predictions are an improvement on just using the raw presence points from a database alone. The outputs can also be used as part of a multidisciplinary approach to inform risk based disease surveillance activities.

## Introduction

Vector borne diseases are a continuing global threat to both animal and human health. The human health impact of mosquito borne diseases such as malaria and Zika virus have been well documented [1,2], as has the effect on livestock from midge-borne diseases such as bluetongue and zoonotic tick borne diseases such as Crimean-Congo haemorrhagic fever [3,4]. The continuing rise in globalisation and the possibility of future environmental and climate change increase the risk of global spread of these pathogens [5]: growth of global trade and human travel increase the risk of introduction of pathogens [6,7], while a change in environment and/or temperature can increase the risk of competent vector species establishing in a previously inhospitable environment [4,8]. Currently, Europe is experiencing the expansion of a number of invasive mosquito species including the Asian tiger mosquito, *Aedes albopictus* [9]. There is also strong evidence that the previously absent mosquito-borne virus, Usutu virus, is now active across Western Europe [10] and the zoonotic virus, West Nile virus, is repeatedly being introduced from Africa into southern Europe [11].

Rift Valley Fever (RVF) is a zoonotic mosquito borne viral disease that can affect both humans and livestock, predominantly cattle and sheep [12]. The disease is caused by infection with Rift Valley fever virus (RVFV). Outbreaks have historically been restricted to sub-Saharan Africa, where the disease is endemic, although trade in livestock is believed to have introduced the virus into the Arabian Peninsula [13]. In 2018 there have been outbreaks of RVF in Kenya, Uganda, South Africa and Rwanda [14–16]. Although RVF has never been reported in Europe, the emergence of a range of African mosquito-borne viruses outside of the continent suggests that it could have the potential to emerge. While there is limited real world evidence for RVF specific to European species, recent laboratory studies have confirmed that lambs bred in Europe are susceptible to infection with RVFV [17,18] and that mosquito species indigenous to Europe are susceptible to infection with the virus and have the capacity to transmit infection [19,20]. Mathematical modelling has suggested that transmission is also possible based on the distribution of mosquito vectors and livestock in The Netherlands [21]. This suggests that it would be prudent to investigate how the virus might be transmitted in a temperate region such as the United Kingdom (UK).

An important first step in understanding the spread of any disease is knowledge about the ecology of vectors and hosts, in particular their distribution and habitat preferences. Ecological niche modelling and habitat suitability models have been used extensively to predict habitat ranges for a number of species around the world [22,23], including vectors associated with RVF [24]. Such models have also been employed to predict the likely range of infectious diseases, such as Ebola in Africa [25]. Environmental modelling has been used in Europe to predict the impact of climate change [8,26], as well as areas at higher risk of RVFV transmission at a country level including Italy and Spain [27,28]. This approach can be used to inform surveillance and outbreak management. Previous methods have used species distribution to inform risk based surveillance activities, e.g. by combining information on wild bird density and commercial poultry flocks, one model has predicted likely hot-spots for the introduction of avian influenza to the UK, the results of which have informed UK surveillance activities [29,30].

Here, we propose a consensus species distribution modelling framework for suitability of RVF competent mosquitos, based on the ecology of vectors and density of livestock hosts of RVF, and demonstrate the application of this output for risk assessment purposes by using the resulting distributions to produce a spatially explicit risk assessment of the most likely areas for establishment of RVFV in the UK livestock population, i.e. areas where introduction of an RVF infected animal or mosquito would lead to transmission of the virus between local livestock and vector populations. This approach used species distribution models to predict the geographical areas of the UK where vector competent mosquito species for RVF may be present. This is then combined with cattle and sheep density maps to predict the areas of the UK where an introduction of RVF is most likely to lead to establishment in the local livestock and vector populations. Other factors will likely affect the dynamics of RVFV transmission in livestock populations and more sophisticated transmission models should be developed for a more accurate analysis.

## Materials & methods

### Overview

**Fig 1:**
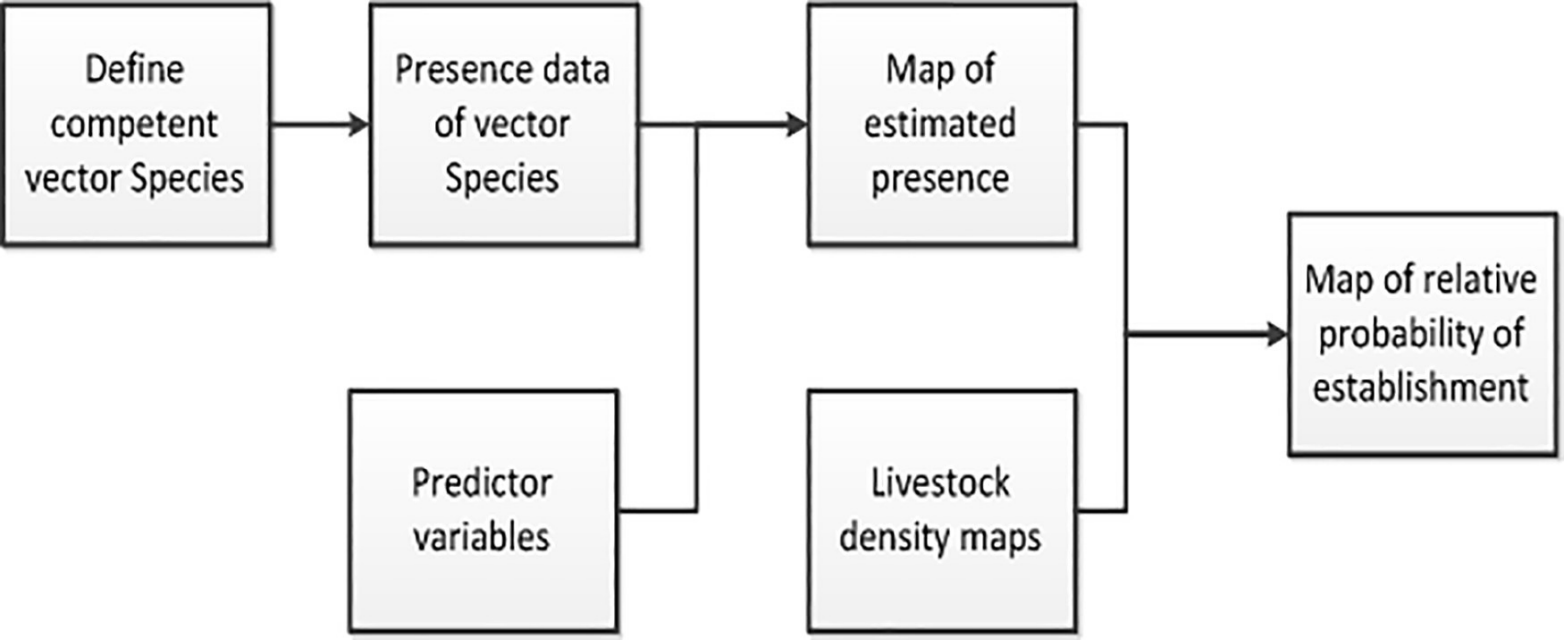
Framework to assess the relative spatial risk of establishment of rift valley fever virus.

The model framework is outlined in Fig 1. Competent vectors for RVFV were identified and georeferenced data of previous observations in the host country were obtained. A list of predictor variables that were considered to be important for the presence of mosquitos was determined, e.g. variables associated with climate and land use. Species distribution models were then used to produce spatially explicit output maps which estimated the relative likelihood of presence of the competent species. The results of all the models were combined to produce one consensus output map representing the suitability for a competent vector species at each location. Finally, the consensus map was combined with cattle and sheep density maps to predict where in the UK an incursion of RVF was most likely to lead to establishment of the virus. We note that this initial risk assessment does not explicitly consider the epidemiology of transmission (e.g. contact rates, basic reproductive number, infectivity, etc.) and that without this our results only highlight locations at risk of high intensity outbreak given an introduction of RVF. The work outlined here, describing vector and host distributions, is a necessary first step to underpin future, more detailed, epidemiological assessments to understand the risk pathway. A more detailed model could assess a definition of establishment based on temporal or spatial dimensions, such as spread to other farms or outbreaks persisting for multiple months, but such complexity was beyond the scope of this assessment.

### Competent vector species

Based on previous studies on mosquito species that have shown evidence of vector competence for RVFV and appropriate species bionomics, potential vectors for RVFV were identified (Table 1) [19,20,31,32]. Vector species were assessed against a number of criteria to define those mosquitoes capable of propagating a disease outbreak. In addition to vector competence for RVFV factors including host-feeding preference, primarily those that feed on livestock, as opposed to birds, were included. Univoltine species were considered less effective vectors due to limited seasonal activity compared to multivoltine species that are active during the whole season [33].

**Table 1:** Table of UK mosquito species bionomics. References are indicated with numerical superscripts: 1 = [34], 2 = [35], 3 = [36], 4 = [37], 5 = [38].

Species^1^	Occurrence in UK^1^	Active (Adults)	Annual Generations (UK)	Feeding Preference
*Species determined to be competent for RVFV*				
***Anopheles claviger***	Widespread	Mar–Oct^3^	Multivoltine^3^	Mammals^3^
***Anopheles messeae***	Widespread	May–Oct^3^	Multivoltine^3^	Mammals^5^
***Anopheles atroparvus***	Widespread	May–Oct^3^	Multivoltine^3^	Mammals^5^
***Culiseta annulata***	Widespread	All year round^2^	Multivoltine^3^	Mammals^4^ /Birds^3^
***Aedes detritus***	Widespread	Mar–Nov^2^	Multivoltine^3^	Mammals^3^
*Species not determined to be competent for RVFV*				
***Aedes cinereus***	Widespread, patchy	June–Aug^2^	Univoltine^1^	Mammals^3^
***Aedes vexans***	Sporadic reports	No Data	No Data	Mammals^3^
***Anopheles algeriensis***	Few reports	No Data	No Data	No Data
***Anopheles daciae***	Few reports	No Data	No Data	No Data
***Anopheles plumbeus***	Widespread	Apr–Oct^2^	Bivoltine^2^	Mammals
***Coquillettidia richiardii***	Widespread	Apr–Aug^2^	Univoltine^3^	Mammals^3^
***Culex modestus***	South East England	Jul–Sept^2^	No Data	Mammals^3^
***Culex pipiens s***.***s***.	Widespread, abundant	Apr–Nov^2^	Multivoltine^3^	Birds^3^
***Culex pipiens***, ***biotype molestus***	Few reports	All year round^2^	Multivoltine^3^	Mammals^3^
***Culex torrentium***	Widespread	Apr–Nov^2^	Multivoltine^3^	Birds^3^
***Culex europeaus***	Few reports	No Data	No Data	No Data
***Orthopodomyia pulcripalpis***	Few reports (SE England)	July–Sept^3^	Univoltine^3^	Mammals^3^
***Culiseta longiareolata***	Few reports	No Data	No Data	No Data
***Culiseta alaskaensis***	Few reports	No Data	No Data	No Data
***Culiseta fumipennis***	Widespread (SE England)	No Data	No Data	No Data
***Culiseta litorea***	Widespread (SE England)	Apr–Aug^2^	No Data	No Data
***Culiseta morsitans***	Widespread	May–Sept^2^	Univoltine^3^	Birds^3^
***Culiseta subochrea***	Rare	No Data	No Data	No Data
***Finlaya geniculatus***	Widespread (England)	No Data	No Data	No Data
***Aedes anulipes***	Widespread	Apr–Sept^3^	Univoltine^3^	Mammals^3^
***Aedes cantans***	Widespread	Apr–Sept^2^	Univoltine^3^	Mammals^3^
***Aedes caspius***	Rare	Apr–Oct^2^	Multivoltine^3^	Mammals^3^
***Aedes communis***	Few reports	Do Data	No Data	No Data
***Aedes dorsalis***	Rare	May–Sept^3^	Multivoltine^3^	Mammals^3^
***Aedes flavescens***	Rare	May–Aug^3^	Univoltine^3^	Mammals^3^
***Aedes leucomelas***	One report	No Data	No Data	No Data
***Aedes punctor***	Widespread	Mar–Oct^2^	Univoltine^3^	Mammals^3^
***Aedes sticticus***	Few reports	No Data	No Data	No Data
***Aedes rusticus***	Widespread	Apr–Sept^2^	Univoltine^3^	Mammals^3^

### Presence/absence of competent vectors

Presence data for indigenous mosquito species listed in Table 1 were obtained from the UK National Biodiversity Network (NBN) [39]. The NBN contains information supplied by a large number of different contributing bodies with varying degrees of accuracy by data providers. To compensate for this we restricted our use to a subset of the NBN data from the National Mosquito Atlas project. Complementary data for absence of mosquito species is not available. However, we used as a proxy, the dataset for Culex mosquitoes (NBN) under the assumption that surveillance at these sites had identified *Culex* spp., it would also have identified other mosquito species if they were present [40].

### Predictor variables

Based on scientific literature and expert opinion, a number of relevant predictor variables were identified. These data predominantly fell into two categories; land cover and climatic factors. The land cover data consisted of 23 ‘target class’ raster files at a 1Km resolution for different UK land cover classes, such as broadleaved woodland, arable and salt marshes, which detailed the proportion of each cell (i.e. 1km^2^) that was of that class [41]. This was considered important as some mosquitos are known to favour specific environments. For example, the mosquito species *Aedes detritus* lays its eggs in weakly saline conditions and is associated with salt marshes, whereas other species breed exclusively in freshwater. The climatic data were obtained from the UK Met Office and consisted of monthly raster files for 11 different climatic variables related to temperature, rainfall, snowfall, sunshine and humidity [42]. We averaged the monthly values to get one annual average raster for each variable. Climatic factors such as temperature are often associated with mosquito activity and breeding [43]. Further description of the predictor variables are provided in the supporting information (S1 File).

### Map of estimated presence

*Species distribution models*: To estimate potential areas of suitable habitat of RVFV competent mosquito species, we developed a consensus model framework, based on an existing methodology that compares a range of different models [22]. It is well established that individual species distribution models have different strengths and weaknesses and can produce different results, due to differences methodologies employed [44–46]. As such, consensus, or ensemble, methods have previously been suggested as ways to combine the individual model results in order to effectively capture the variability between the model results, e.g. [22,47,48]. Here, we first assessed six habitat suitability models using packages available in R [49] (Table 2). They were; bioclim, multiple generalised linear model (glm), support vector machines (svm), Maximum Entropy (MaxEnt), boosted regression trees (brt) and Random Forest (rf).

**Table 2:** List of models, description and reference.

Method	Description	Functions available from
*Bioclim*	Classic 'climate-envelope-model'	https://www.rdocumentation.org/packages/dismo/versions/1.1-4/topics/bioclim
*MaxEnt*	Maximum entropy, machine learning algorithm	https://cran.r-project.org/web/packages/maxent/maxent.pdf
*BRT*	Boosted regression trees, machine learning method	https://cran.r-project.org/web/packages/gbm/gbm.pdf
*RF*	Random forest, machine learning method	https://cran.r-project.org/web/packages/randomForest/randomForest.pdf
*SVM*	Support vector machines, supervised learning method	https://www.rdocumentation.org/packages/e1071/versions/1.6-8/topics/svm
*GLM*	Multiple generalised linear regression model.	https://cran.r-project.org/web/packages/glmulti/glmulti.pdf

*Inputs*: The presence data and predictor variables were loaded into the R statistical package. The predictor variable raster files were reformatted to be in the same projection and resolution, normalised to the same scale, and then combined into one raster stack. To account for sample selection bias [40], the background/absence points were selected based on 1km raster cells where other *Culex* spp. mosquitos had been found, but not the species we consider competent for RVF; it is possible *Culex spp*. and RVF competent mosquitos could be observed in the same cell, but this would not be considered an absence. A subset of these points were selected at random, in order for the number of background points used in the model to equal the number of presence points [50]. As we are considering the whole 1km raster cell, rather than the specific point, each observation, either presence or absence, can be considered independent with regards to different environmental conditions. To provide an independent dataset to evaluate the predictive accuracy of the models, the presence and background points were then randomly grouped into training and testing datasets. A subset of the data (20%) were reserved for testing, using the *kfold* function from the dismo package in R [51]. To account for the variability introduced by the random selection of background (absence) locations and the partitioning of data for testing and training this process was performed 100 times fitting the set of models each time.

*Collinearity*: To test for collinearity between predictor variables, the *vifstep* function in the *usdm* package in R was used [52], with predictor variables failing the test removed from the analysis for that iteration. Then the different algorithms were run using these data as input variables.

*Consensus model*: Each species distribution method has an associated *threshold* function in the *dismo* package of R. This function calculates a cut-off value to transform the model predictions into a binary presence/absence score, i.e. one would realistically expect presence of the vector in areas where the model output value is above this threshold value and absence of the vector in areas below the threshold value. We chose to base the cut-off on the point at which the sum of the sensitivity and specificity is highest. The consensus model method used the threshold calculations from all the individual models to determine which areas were consistently flagged as being above the threshold by multiple models. For each species distribution model, *i*, and iteration, *q*, a threshold map was produced where each geographical cell, *j*, had a binary value, *h*_*0*_*(i*,*j*,*q)*, to denote either presence (1) or absence (0)

$$h_{0}(i,j,q)=\left\{ \begin{array}{cc} 0 & g(i,j,q)<t(i,j,q)\\ 1 & g(i,j,q)\geq t(i,j,q) \end{array},\right.$$

where *g(i*,*j*,*q)* is the value of species distribution model *i* at cell *j* in iteration *q* and *t(i*,*j*,*q*) is the corresponding threshold value. The sum of these values over all iterations, *h(i*,*j)*, represent how often the value in that cell is above the threshold value; the higher the score, the more likely it is the vector is present in that cell.

$$h(i,j)=\sum_{q=1}^{Q(i)}h_{0}(i,j,q),$$

where *Q(i)* is the number of iterations for model *i* used in the consensus model. This resulted in six maps, one for each method. The six maps were then combined to produce the consensus model map of estimated probability of presence, with the value in cell *j*, *m(j)*, given by

$$m(j)=\frac{\sum_{i=1}^{N_{sdm}}h(i,j)/Q(i)}{N_{sdm}},$$

where *N*_*sdm*_ was the number of individual species distribution models.

### Establishment of RVF

The species distribution maps predict where in the UK we can expect to find mosquito species capable of transmitting RVF, but they reveal little about RVFV transmission dynamics, such as where it is most likely to occur, spread and cause a large and/or prolonged outbreak. To demonstrate how these maps can be incorporated into a wider model to address such issues, a simple assessment to predict where establishment of RVF in the UK is most likely was conducted. The consensus model raster map for the suitability of competent mosquito species was combined with factors thought to be necessary for establishment of RVF, in this case maps of cattle and sheep density [23,53]. A compentent animal host resevoir is essential for transmission and thus establishment of RVF. Livestock density is critical as the infectious period is short for RVFV infected animals after which the animal is unlikely to be infected [21]. A small host population would become infected and seroconvert rapidly, reducing the likelihood of establishment and a prolonged outbreak. Whilst it is possible other animal species, for example camels, may also be competent hosts, cattle and sheep are the main livestock species present in the UK that are commonly linked with RVF outbreaks. For the final risk map, we capped the livestock densities at a value of 100, under the assumption that the risk would be the same for any numbers above this level. Thus the risk of establishment in cell *j*, *R(j)*, was given by

R(j)=(c(j)+s(j))*m(j),

where *c(j)* and *s(j)* are the cattle and sheep densities in cell *j* (capped at 100), and *m(j)* is the consensus map output value in cell *j*.

### Model validation

*Goodness of fit*, *environmental data*: An important consideration with regards to the validity of the model to the whole of the UK is whether the environmental conditions of a particular area are well represented in each iteration of the model. For example, if regions 1000m above sea level are rarely selected in the training data, then the model predictions for these regions will not be as reliable as the predictions outside the domain of the model. To assess this we use the Multivariate Environmental Similarity Surfaces (MESS) algorithm in the R *dismo* package [44], to compare the regions selected in the training data to the regions in the full dataset, i.e. the whole of the UK. Negative values for the MESS statistic indicate regions of the UK that are not well represented in the training dataset. Each iteration the MESS algorithm gives a value for each 1km cell in the UK. Each cell was then assigned the value 0 if the MESS statistic is negative and 1 if positive. These were then aggregated over all iterations to produce a single map.

*Goodness of fit*, *consensus model*: The goodness of fit of the final consensus model was estimated by considering four statistics; a) the Area Under the receiver operator Curve (AUC), b) the correlation coefficient, c) the average model predicted probability of presence at the observed presence points, d) the average model predicted probability of absence at the observed absence points. Statistics c) and d) were calculated simply by extracting the values of the consensus model at the observed presence and absence points. The correlation statistic was calculated using the *cor*.*test* function in R, comparing the model predicted values at presence and absence points against a statistic that took the value of 1 for presence points and 0 for absence points [54]. The AUC was estimated by assuming equivalence to the Wilcoxon rank sum test statistic first calculating the Mann-Whitney-Wilcoxon U statistic [55]. This was calculated by running the *wilcox*.*test* R function on the model values at the observed presence and absence points, then dividing the resulting statistic by the total number of observed presence and absence points [54].

*Consensus model individual method selection*: To assess the validity of including all the individual models in the consensus model, we fit alternative models, each removing one of the individual models, i.e. we fit six alternative models using only five of the species distribution models. These alternative models were compared to the full consensus model using the same goodness of fit statistics.

*Predictor variables*: As well as the collinearity analysis conducted in the main model framework, we also conducted an ANOVA analysis to assess the relationship between the predictor variables and the model values. This analysis uses a methodology described previously [56]. Briefly, the values of each predictor variable are ranked from low to high and assigned a semi-quantitative score based on quartiles (the lowest quarter of values are assigned the value 1 and the highest the value 4). A linear model is then fit which regresses the consensus model values against the transformed predictor variables. The resulting F values, one for each predictor variable, provide a measure of the extent to which the model values and the predictor variable are related. Individual linear models are also fit, with each predictor variable regressed against the model outputs. The sign of the gradient coefficient is used to determine the direction of the relationship. Note that this analysis does not indicate whether the predictor variables should be included or not, this is done in the collinearity assessment within the model framework, a variable with a high F value may not be necessary if other predictors can act as proxy for it.

### Outputs

The outputs of the individual species distribution models were raster files at a 1Km spatial resolution that represent the relative habitat suitability of the competent mosquito species for the six different models. Each model method produces 100 potential distribution maps, for the different partitions of test and training data. Maps are produced for the mean values over all iterations for each method, along with the 5^th^, 50^th^ and 95^th^ percentiles of the goodness of fit statistics. The output of the establishment model is a raster file at 1Km spatial resolution that represents the relative probability of establishment of RVF across the UK.

## Results

**Fig 2:**
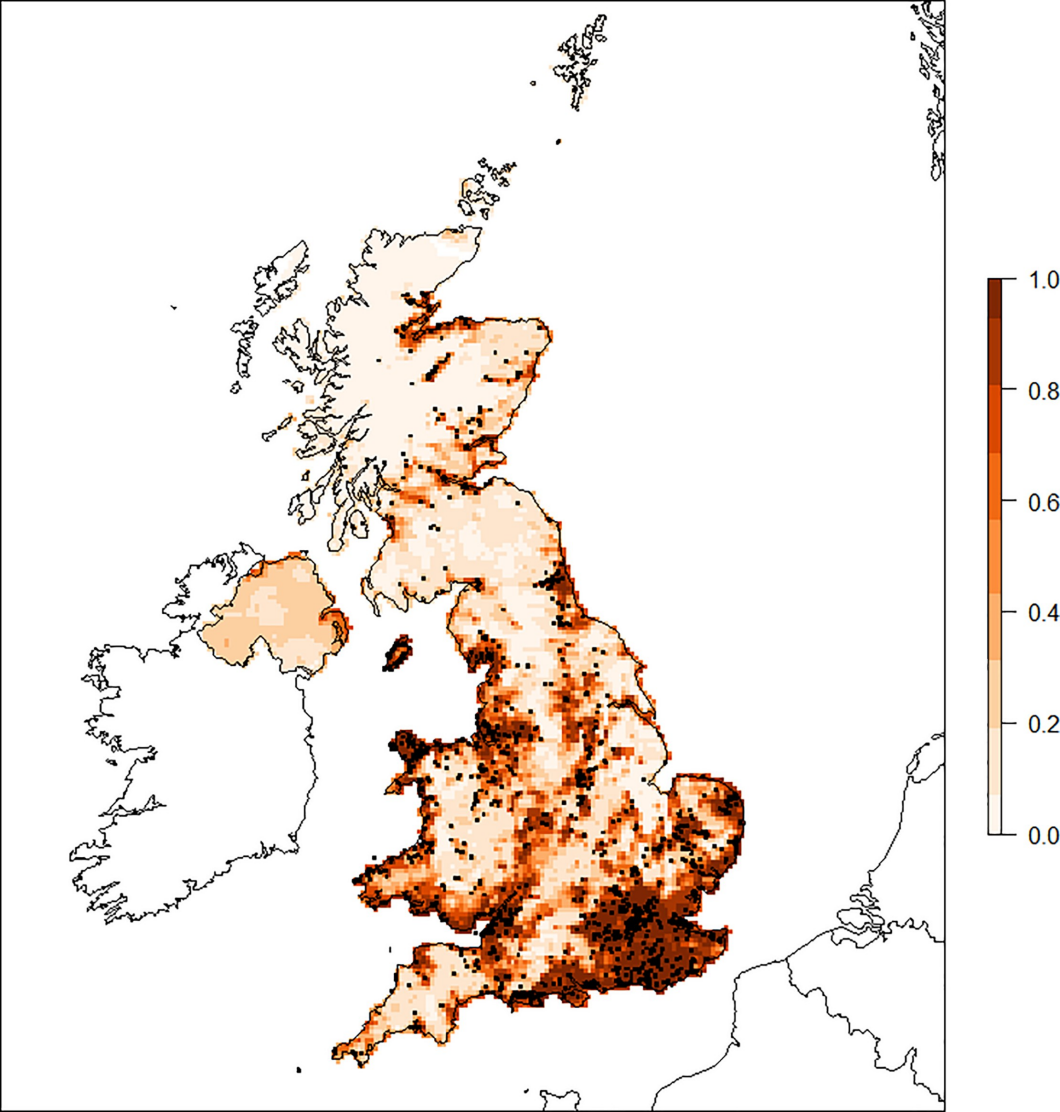
Consensus model map indicating probability of presence of RVF competent mosquito species. Higher values closer to 1 (darker shade) indicate higher relative probability of presence of competent mosquito species compared to lower values. Observed mosquito presence data are shown by the black squares.

*Consensus model*: Fig 2 shows a map of the consensus mosquito suitability model, detailing areas of the UK which were consistently identified as being above the threshold value by multiple models (as shown by the darker red colour). The presence points used in the model fits were also plotted over the map (black dots). It can be seen that the South East of England, including areas around London, are consistently identified, along with areas around Suffolk and Norfolk, and many coastal regions, including as far north as Inverness on the east coast of Scotland. Areas where competent vector species have been recorded tended to be above the threshold value in most iterations of the individual models, and hence have a high value in the consensus model. However, areas where there are only a few observations nearby were not consistently above the threshold, particularly in the more rural areas of Scotland. A plot including the absence points (see supporting information, S1 File) shows that *Culex spp*. have been observed throughout the UK (except for Northern Ireland where we had no presence or absence points), including areas the model predicts to be of low probability. This suggests that the results are not biased by a lack of sampling in a particular region, based on the assumption that observing at least one *Culex* species in a region and not observing any of the species we consider competent for RVF is a good indication of absence of these species. The individual maps from each method are provided in the Supporting information (S1 File).

**Fig 3:**
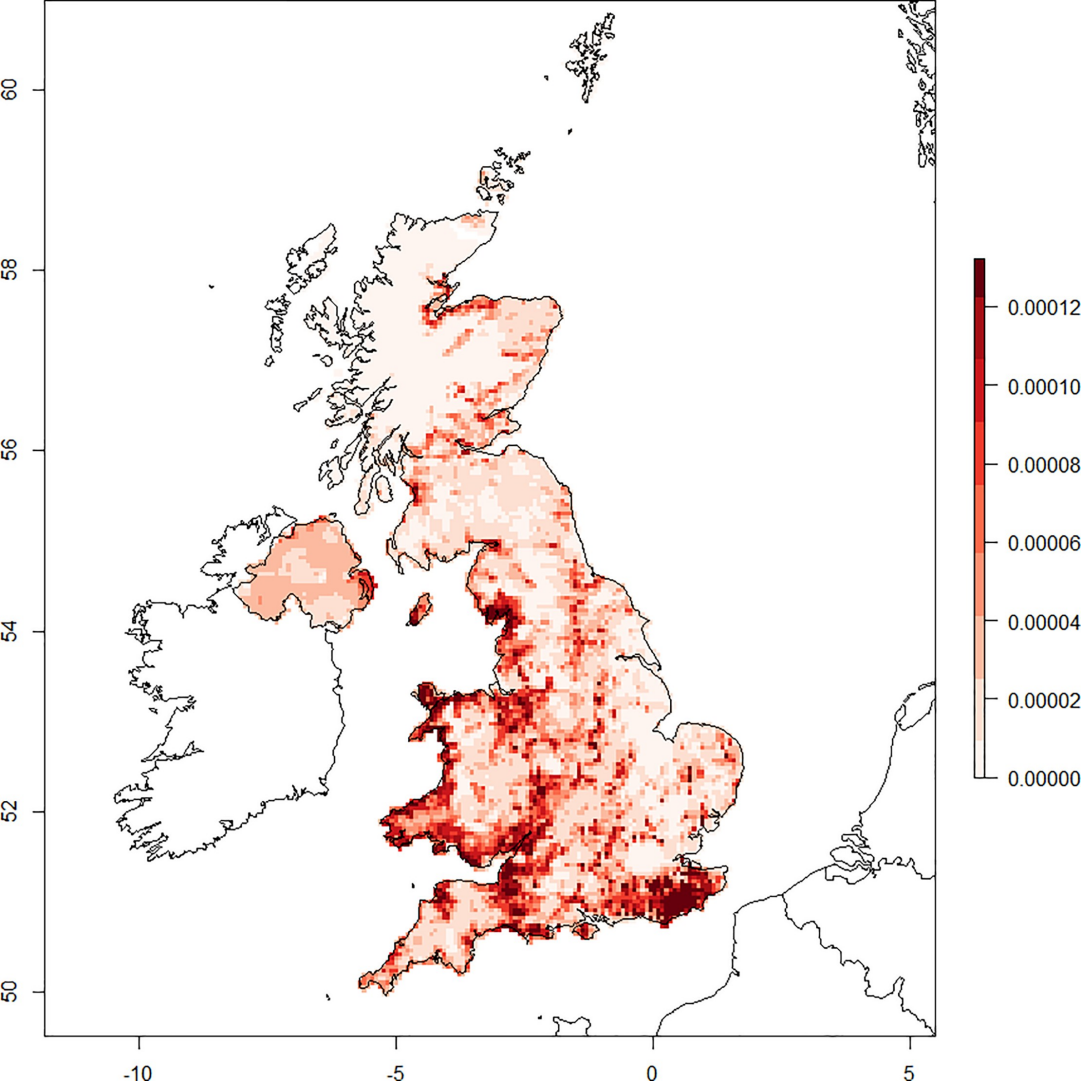
Risk of establishment of RVF. Higher values (darker shade) indicate higher relative probability of establishment in UK, given entry of RVF has occurred.

*Risk of establishment*: The simple estimation of the relative risk of establishment of RVFV suggests that while there is a high probability of presence of competent mosquito species in the South East of England, the lower livestock densities mean that there is a lower risk of establishment than other areas such as in the South West and the coasts of Wales (Fig 3).

**Fig 4:**
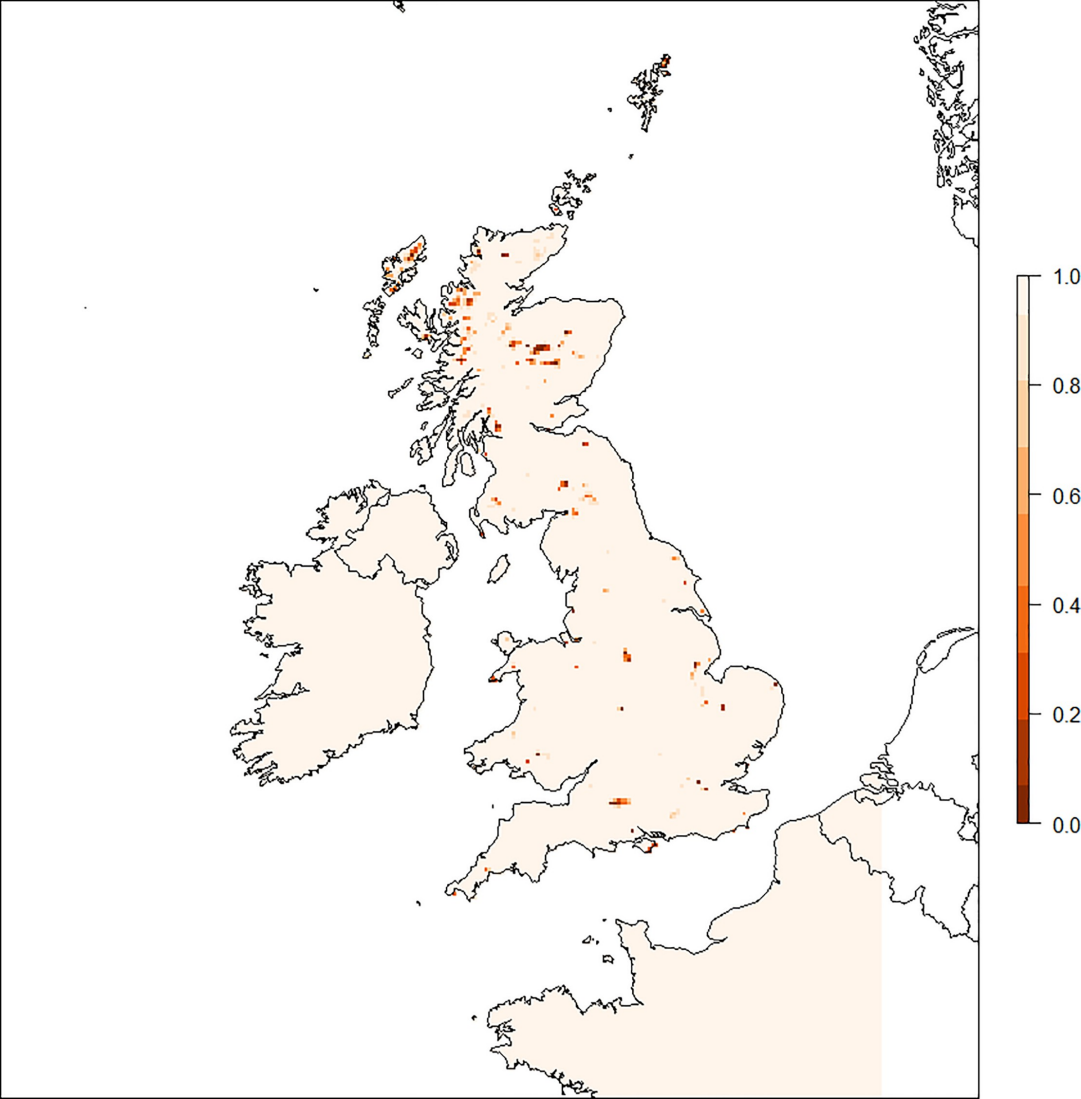
UK map detailing the results of the MESS analysis. The map shows the averaged result over all iterations of the model; a value close to 1 (lighter) indicates the region is described well by the training data, while a value close to zero (darker) indicates it is not.

*Goodness of fit*, *environmental data*: The UK map of the MESS statistic suggests that in general the UK environmental conditions are well represented by the training data, but there are a number of small regions around the UK where the training data is not always fully representative, for example in some parts of the highlands of Scotland (Fig 4).

*Goodness of fit*, *consensus model*. There was a clear difference in the model predicted suitability at locations where RVF competent mosquitos had been observed, as opposed to where they are expected to be absent (Table 3). There was high variability in these values, the 5^th^ percentile model value at the presence points was 0.388, but the AUC value was estimated to be 0.883 (a value above 0.7 is generally considered a good fit). Analysis suggested that there was not a huge impact on these goodness of fit values if individual species distribution models were removed from the consensus model (Table 3). The biggest noticeable difference was that the average value at the absence points decreased when the bioclim method was removed. However, the average value at the presence points also decreased, suggesting that while the probability of a false positive might be reduced, the probability of a false negative increased.

**Table 3:** Goodness of fit statistics for the full consensus model and consensus models without individual models. Statistics that are better compared to the full consensus model are shaded in grey.

Method	Correlation	Area under the curve (AUC)	Mean model value at presence points (5^th^,95^th^ percentiles)	Mean model value at absence points (5^th^,95^th^ percentiles)
Full Model	0.280	0.882	0.845 (0.388,1)	0.287 (0,0.939)
No bioclim	0.296	0.892	0.841 (0.319,1)	0.219 (0,0.941)
No glm	0.290	0.893	0.867 (0.462,1)	0.295 (0,0.932)
No svm	0.281	0.886	0.858 (0.460,1)	0.302 (0,0.932)
No MaxEnt	0.283	0.886	0.849 (0.433,1)	0.296 (0,0.934)
No Brt	0.264	0.870	0.830 (0.363,1)	0.302 (0,0.949)
No Rf	0.253	0.859	0.826 (0.314,1)	0.310 (0,0.965)

**Fig 5:**
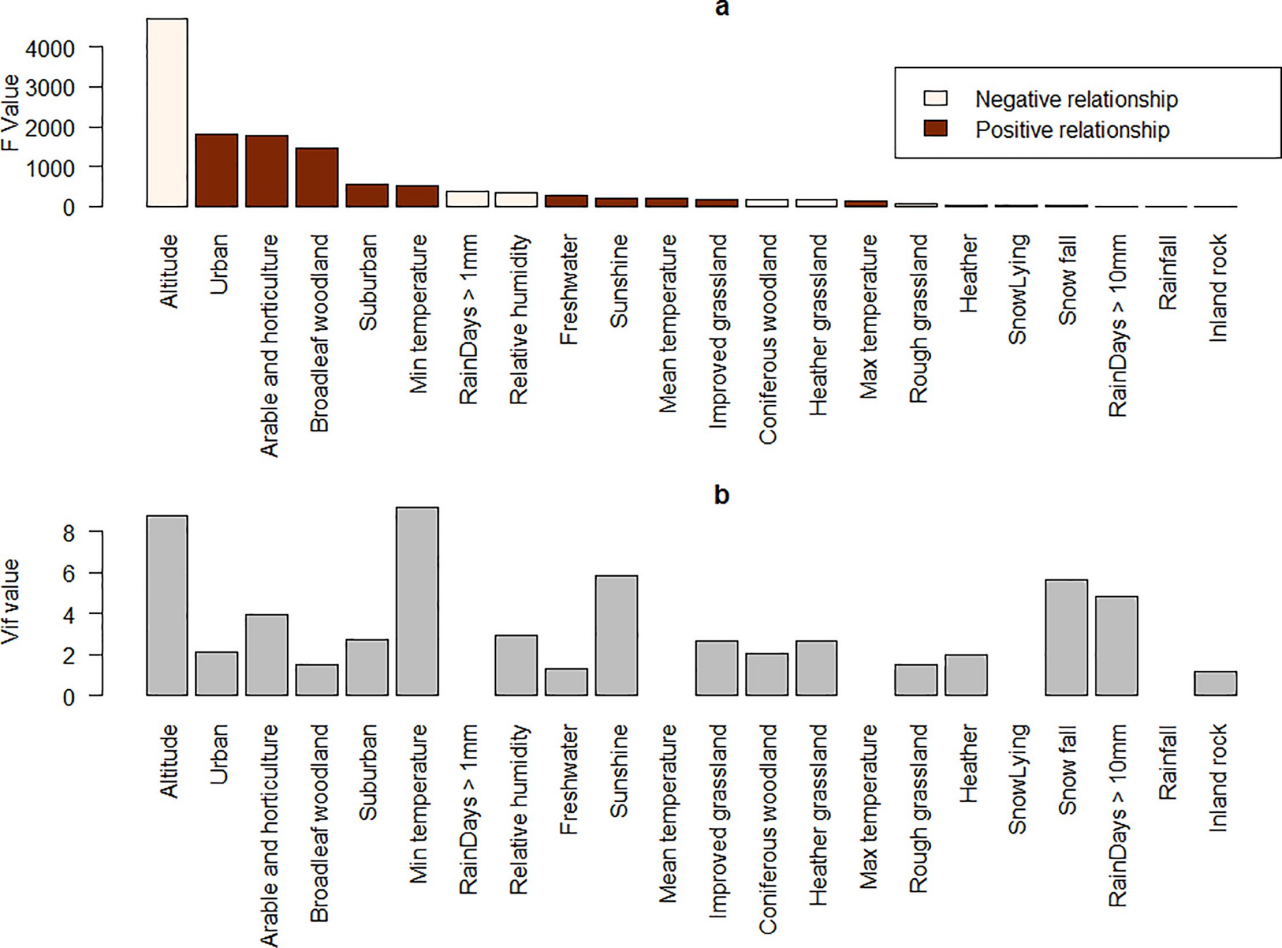
ANOVA analysis F value indicating relative importance of model predictors on consensus model values. (Fig 5A) Colour indicates direction of relationship between predictor and output. (Fig 5B) VIF values for model predictors, higher values indicate predictor less likely to be needed in the model, a value of 0 indicates that the variable was never chosen in the model.

*Predictor variables*: The ANOVA analysis suggests that altitude, urban, arable and horticultural land have the largest impact on the model outputs, i.e. the spatial variability in these predictors influenced the spatial variability in the model outputs. Additionally, RVF competent mosquitos are less likely to be present at high altitude and on arable and horticultural land as these predictors have negative coefficients in fitted linear models, but were more likely to be present on urban or broadleaf woodland as these predictors had positive coefficients (Fig 5). Altitude had a relatively high vif value indicating relative high collinearity with other predictor variables; in some iterations the value exceeded 10 and so was removed from the model. However, in general the selection of the test and training data has limited impact, with most predictors either always included (*vif*<10) or always excluded (*vif*>10).

A scenario was run including additional predictor variables related to seasonality of temperature and precipitation, such as the minimum temperature of the coldest month and precipitation of the wettest quarter. These variables were calculated from the same Met Office data used for the baseline climate variables. While the model did select some of these variables, their inclusion was found to have little impact on the consensus model output; there was no noticeable visual difference and only an increase of 0.005 in the AUC value. Further details of this analysis can be found in the supplementary information.

## Discussion

The study presented here demonstrates the application of habitat suitability modelling and host distribution to predict hot spots for establishment of RVF. The results suggested that less than half of the predictor variables influenced the model outputs. The negative relationship between altitude and establishment risk and the positive relationship between temperature and establishment risk are a result of many mosquito species being inactive below certain temperatures. However, it is not clear if the positive relationship associated with urban and suburban areas is due to a specific factor or if it is a proxy for another undefined factor, such as reduced density of other animal species that the mosquitos might preferentially feed on. The vif test used to analyse the input data removed the maximum and average temperature predictors, presumably due to their correlation with the minimum temperature predictor.

The approach was also used to analyse habitat suitability of individual mosquito species, but due to a lack of observed data for some species the results were considered highly uncertain. A useful extension of this work would be to estimate mosquito abundance as well as determine their presence. However, whilst there are a number of small-scale studies evaluating mosquito abundance [57], there are currently little available data across large regions of the UK. Additionally, mosquito abundance is dependent on many factors including temperature, species seasonality and availability of larval habitat. It would be useful if abundance data could be standardised, collated and stored in one place, perhaps alongside the presence data on a site such as the National Biodiversity Network or the Global Biodiversity Information Facility (GBIF) [41].

The consensus model combines results from six different species distribution models. Each method was assessed multiple times (iterations) to account for differences in outputs due to selection of background data. Visual inspection of output maps indicated that differences between iterations were small, but particularly apparent when test data points are selected from areas with no other points geographically close to use for training.

By using the threshold values from each method to produce the final consensus output we avoided issues of different scales of the underlying values which could be an issue if just taking the average of the original values. However, the threshold value is to some extent subjective and we do lose information about the relative likelihood for very low and very high probability areas. While there may be differences in goodness of fit between the individual species distribution models, goodness of fit statistics on the consensus model suggest that there is little improvement from dropping any of the individual models from the consensus model (Table 3).

A potential refinement to this methodology could be to omit specific iterations if they do not meet certain goodness of fit criteria. For example, inclusion of an AUC that is significantly lower than the average for that method. However, there are many different ways to assess the goodness of fit of these models and there is no universally accepted ‘gold standard’ method to achieve this. The analysis presented here (Table 3), demonstrates that a model can perform less well by one metric, e.g. probability of presence at observed presence points, but better by another metric, e.g. probability of absence at observed absence points. Additionally, it is important to distinguish between what is an unrealistic poor model fit and what could still be a potentially biologically plausible, if relatively unlikely, result given the input data. For example, we have estimated our absence data based on where our RVF competent mosquito species have been not been observed, but other *Culex* species have. This is not confirmation of the absence of RVF competent mosquito species in these areas, so there is uncertainty associated with assessing the species distribution models based on predicting low probability of presence in these areas. As such, we believe the consensus approach used here, combining results from multiple iterations of multiple models, is a good approach. It does not lose any of the information by omitting specific models or iterations and reduces some of the concern regarding poor fitting; if one method or iteration is significantly different to the others, the impact on the consensus model will be down weighted. If, on a case by case basis, there are some clear criteria for omitting certain iterations then this could be incorporated into the methodology.

A scenario including predictor variables that better account for seasonality was run. While this could be thought to be particularly important given the inherent seasonality in RVF transmission, there was very limited impact on the consensus model results. However, we do consider that it would be good practice to consider whether they should be included in other models on a case by case basis, e.g. if considering a similar approach for another pathogen.

The challenges and assumptions inherent in niche modelling, mean that the output maps should not be considered to be a completely accurate prediction of presence. However, we believe that this approach is an improvement over reliance on using only the raw presence points as input for mathematical models and risk assessments; by only using the raw data presence points for competent mosquitos to inform a risk assessment with no further analysis, absence would be assumed in all areas with no reported observations. We demonstrate this with a simple spatial model that can give an indication of UK geographical areas that are more at risk of establishment of RVF than others. Other factors will likely affect the dynamics of RVFV transmission in livestock populations and more sophisticated transmission models should be developed for a more accurate analysis. Within-herd factors such as biting rates and livestock movement could be important in promoting disease spread. Furthermore, factors that could affect the gonotrophic cycle of mosquitos, such as temperature or rainfall, will also influence virus transmission, as a shorter mosquito life-cycle could mean mosquitos biting livestock more frequently, giving more opportunity for transmission.

The methodology proposed here, both the consensus model and risk of establishment, can theoretically be adapted for other disease with different competent vector species, such as those mosquito species competent for malaria, Zika or dengue fever, and for other geographical regions, such as the whole of Europe, if data are available. However, this would need to be assessed on a case by case basis as factors such as sample size and the complexity of the interactions of the predictor variables can reduce the predictive accuracy. The outputs of this work, can be used as part of a multidisciplinary approach to inform risk based disease surveillance activities.

## Supporting information

S1 File Supporting information, predictor variables and additional maps for the individual species distribution models. (DOCX)
