# Supplementary material for: Using species distribution models to predict potential hot-spots for Rift Valley Fever establishment in the United Kingdom
Source: PLoS One. 2019 Dec 23;14(12):e0225250. doi: 10.1371/journal.pone.0225250 (PMC6927579; doi:10.1371/journal.pone.0225250)
Supplement: S1 File — (DOCX) [file pone.0225250.s001.docx]

# Supporting information

## Predictor Variables

Table S1 details the predictor variables with additional description for the climatic variables, along with additional information from the MaxEnt, Boosted Regression Trees and Random Forest methods regarding their importance in the models.

Table S1: Table of vif values for predictors (5th, 50th and 95th percentiles), the number of times the predictor is included in the analysis, *N*, (i.e. vif<10) and the average rank of the predictor variables over 100 iterations for various statistics regarding the relative importance of the predictor variables (1 is most important) from the MaxEnt, Random Forest and BRT models. Note factors that were never included have NA values.

| Variable | N | Vif: 5% | Vif: 50% | Vif: 95% | MaxEnt: Contribution | MaxEnt: Permutation  importance | Brt: Relative influence | Random Forest: MSE | Random Forest:  Node Purity |
| --- | --- | --- | --- | --- | --- | --- | --- | --- | --- |
| Suburban | 100 | *2.72* | *2.84* | *3.02* | 1 | 1.02 | 2.19 | 1.92 | 1.92 |
| MinTemp: Average of the daily lowest air temperatures from 0900-0900 (°C) | 100 | *8.54* | *8.89* | *9.21* | 2 | 13.03 | 1 | 1.09 | 1.09 |
| Arable and horticulture | 100 | *3.96* | *4.14* | *4.38* | 6.39 | 5.63 | 5.85 | 5.4 | 5.4 |
| Urban | 100 | *2.08* | *2.16* | *2.24* | 6.6 | 14.76 | 3.46 | 3.11 | 3.11 |
| Sunshine: Duration of bright sunshine during the month (hours per day) | 100 | *5.29* | *5.51* | *5.72* | 8.53 | 6.48 | 8.66 | 4.72 | 4.72 |
| RelativeHumidity: Hourly (or 3-hourly) relative humidity (%) averaged over the month | 100 | *3.05* | *3.19* | *3.34* | 5.77 | 5.47 | 4.09 | 9.04 | 9.04 |
| Improved grassland | 100 | *3.64* | *3.77* | *3.91* | 3.94 | 2.38 | 11.24 | 8.75 | 8.75 |
| Broadleaf woodland | 100 | *1.54* | *1.57* | *1.60* | 9.41 | 6.83 | 6.69 | 7.97 | 7.97 |
| SnowFall: Number of days with sleet or snow falling | 100 | *5.39* | *5.67* | *5.98* | 14.60 | 7.21 | 8.41 | 5.07 | 5.07 |
| Rough grassland | 100 | *1.53* | *1.58* | *1.63* | 11.52 | 9.82 | 12.69 | 16.64 | 16.64 |
| Fen marsh and swamp | 100 | *1.07* | *1.09* | *1.10* | 6.21 | 15.91 | 13.55 | 17.95 | 17.95 |
| Coniferous woodland | 100 | *2.00* | *2.07* | *2.14* | 17.94 | 12 | 15.12 | 15.22 | 15.22 |
| Bog | 100 | *1.70* | *1.77* | *1.83* | 9.79 | 11.33 | 22.3 | 17.47 | 17.47 |
| Inland rock | 100 | *1.12* | *1.15* | *1.22* | 16.35 | 13.85 | 13.03 | 20.07 | 20.07 |
| Acid grassland | 100 | *3.43* | *3.56* | *4.68* | 25.08 | 21.02 | 20.55 | 9.91 | 9.91 |
| RainDays10: Number of days with ≥10 mm precipitation (0900-0900) | 100 | *4.54* | *4.74* | *4.93* | 25.57 | 24.26 | 13.8 | 12.27 | 12.27 |
| Heather grassland | 100 | *2.43* | *2.53* | *2.60* | 24.22 | 20.78 | 19.43 | 13.16 | 13.16 |
| Heather | 100 | *1.82* | *1.87* | *2.05* | 22.83 | 17.79 | 21.51 | 16.3 | 16.3 |
| Freshwater | 100 | *1.24* | *1.26* | *1.29* | 19.26 | 20.44 | 18.43 | 20.01 | 20.01 |
| Neutral grassland | 100 | *1.22* | *1.24* | *1.33* | 24.55 | 25.98 | 16.75 | 18.19 | 18.19 |
| Littoral sediment | 100 | *1.84* | *1.97* | *2.10* | 15.51 | 23.03 | 22.14 | 24.03 | 24.03 |
| Supra littoral sediment | 100 | *1.16* | *1.18* | *1.21* | 17.16 | 20.98 | 27.59 | 27.09 | 27.09 |
| Saltmarsh | 100 | *1.33* | *1.41* | *1.53* | 19.48 | 26.39 | 24.82 | 24.88 | 24.88 |
| Montaine habitats | 100 | *2.28* | *2.44* | *3.45* | 21.51 | 12.26 | 30.16 | 29.73 | 29.73 |
| Saltwater | 100 | *1.52* | *1.57* | *1.67* | 27.7 | 28.48 | 25.82 | 23.35 | 23.35 |
| Calcareous grassland | 100 | *1.01* | *1.02* | *1.02* | 26.85 | 24.32 | 24.87 | 26.67 | 26.67 |
| Altitude | 19 | *9.69* | *9.89* | *9.97* | 27.38 | 25.88 | 26.85 | 26.15 | 26.15 |
| Littoral rock | 100 | *1.20* | *1.23* | *1.26* | 25.29 | 26.28 | 27.89 | 27.81 | 27.81 |
| Supra littoral rock | 100 | *1.15* | *1.18* | *1.22* | 29.07 | 26.63 | 28.79 | 29.65 | 29.65 |
| MaxTemp: Average of the daily highest air temperatures from 0900-0900 (°C) | 0 | *NA* | *NA* | *NA* | *NA* | *NA* | *NA* | *NA* | *NA* |
| MeanTemp: Average of mean daily maximum and mean daily minimum temperatures (°C) | 0 | *NA* | *NA* | *NA* | *NA* | *NA* | *NA* | *NA* | *NA* |
| RainDays1: Number of days with ≥1 mm precipitation (0900-0900) | 0 | *NA* | *NA* | *NA* | *NA* | *NA* | *NA* | *NA* | *NA* |
| Rainfall: Total precipitation amount (mm) during the month | 0 | *NA* | *NA* | *NA* | *NA* | *NA* | *NA* | *NA* | *NA* |
| SnowLying: Number of days with greater than 50% of the ground covered by snow at 0900 | 0 | *NA* | *NA* | *NA* | *NA* | *NA* | *NA* | *NA* | *NA* |

To assess which predictor variables best define suitability of RVF competent mosquitoes, we compared the distributions of non-zero values of the predictor variables, including the cattle and sheep density, in cells where the consensus model score was greater than 0.95 and cells where it was less than 0.05 (Fig S1). We also compared the two distributions using a Kolmogorov-Smirnov test, the null hypothesis being that the two distribution come from the same underlying distribution (Table S2). Thus, if the null hypothesis is rejected then there is evidence of a statistically significant difference in the predictor variable values between areas suitable for RVF competent mosquitoes and areas not suitable. This analysis, suggested that most of the variables had significantly different distributions at the 1% level (i.e. p < 0.05). For a number of distributions this was due to differences in the number of zero values as a similar test suggested there was no significant difference in the non-zero values, although for some of these variables there were a limited number of non-zero values which may affect the validity of this result. Areas considered suitable for RVF mosquitos tended to have a higher maximum temperature, less snow fall.


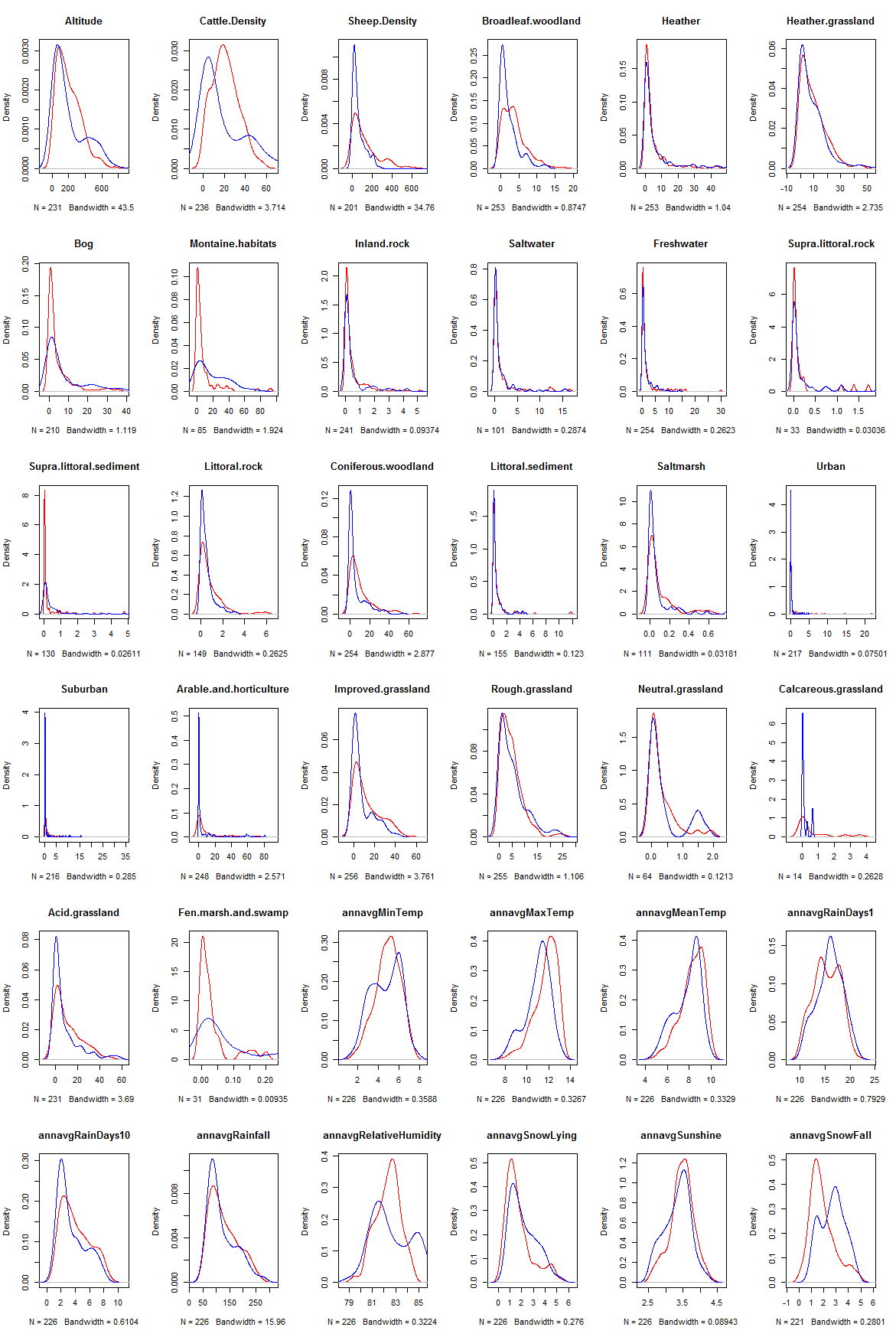


*Figure S1: Density plots of the non-zero values of the predictor variables, in cells where the consensus model is greater than 0.95 (red line) and less than 0.05 (blue line).*

*Table S2: Summary statistics and Kolmogorov-Smirnov (ks) test p value for comparison of the distributions of the values of the predictor variables (with and without zeros included) in cells where the consensus model was >0.95 and <0.05. The lower the ks value the more different the distributions, values where distributions are not significantly different at the 1% level highlighted in red.*

| Predictor Variable | Mean predictor value in cells where model  > 0.95 | Mean predictor value in cells where model  < 0.05 | Kolmogorov-Smirnov test p value | Kolmogorov-Smirnov test p value (non-zero values only) |
| --- | --- | --- | --- | --- |
| Broadleaf woodland | 1.4328 | 0.2044 | 0.0000 |  |
| Heather | 1.7355 | 0.7456 | 0.0000 | 0.0000 |
| Heather grassland | 3.5894 | 1.1219 | 0.0000 | 0.6626 |
| Bog | 1.3633 | 1.0586 | 0.0000 | 0.0461 |
| Inland rock | 0.1640 | 0.1145 | 0.0000 | 0.0121 |
| Freshwater | 0.6536 | 0.1737 | 0.0000 | 0.0309 |
| Coniferous woodland | 4.0467 | 0.7493 | 0.0000 | 0.7223 |
| Urban | 0.2444 | 0.0269 | 0.0000 | 0.0000 |
| Suburban | 0.6917 | 0.0620 | 0.0000 | 0.0204 |
| Arable and horticulture | 4.0035 | 0.7845 | 0.0000 | 0.0000 |
| Improved grassland | 4.8969 | 1.0686 | 0.0000 | 0.0000 |
| Rough grassland | 1.8003 | 0.7092 | 0.0000 | 0.0000 |
| Acid grassland | 3.9387 | 0.9911 | 0.0000 | 0.0892 |
| Sheep Density | 31.6661 | 5.0702 | 1.4433E-15 | 0.0000 |
| SnowFall | 1.8865 | 2.6951 | 9.3592E-14 | 0.0001 |
| Cattle Density | 21.2387 | 21.5504 | 5.0707E-12 | 0.0000 |
| MaxTemp | 11.7219 | 10.9917 | 6.0475E-10 | 0.0000 |
| Littoral sediment | 0.1501 | 0.0418 | 1.4181E-09 | 0.0000 |
| Littoral rock | 0.1841 | 0.0447 | 9.4014E-08 | 0.9317 |
| Supra littoral sediment | 0.0568 | 0.0433 | 2.7580E-06 | 0.0289 |
| Saltmarsh | 0.0166 | 0.0056 | 5.1725E-06 | 0.0000 |
| RelativeHumidity | 82.3363 | 82.6479 | 1.6817E-05 | 0.1562 |
| annavgRainDays10 | 4.0696 | 3.4567 | 0.0002 | 0.0000 |
| MeanTemp | 8.2880 | 7.8340 | 0.0002 | 0.0002 |
| SnowLying | 1.7399 | 2.1492 | 0.0007 | 0.0002 |
| Montaine habitats | 1.0004 | 0.7830 | 0.0021 | 0.0007 |
| Altitude | 199.8442 | 195.9276 | 0.0030 | 0.0000 |
| Neutral grassland | 0.0304 | 0.0061 | 0.0033 | 0.0030 |
| annavgRainDays1 | 15.3671 | 15.9962 | 0.0061 | 0.8040 |
| Sunshine | 3.4626 | 3.3332 | 0.0087 | 0.0061 |
| MinTemp | 4.9158 | 4.7122 | 0.0131 | 0.0087 |
| Saltwater | 0.2008 | 0.0984 | 0.0139 | 0.0131 |
| Rainfall | 128.5561 | 120.9969 | 0.0703 | 0.6314 |
| Supra littoral rock | 0.0087 | 0.0069 | 1 | 0.0703 |
| Calcareous grassland | 0.0134 | 0.0012 | 1 | 0.8419 |
| Fen marsh and swamp | 0.0016 | 0.0050 | 1 | 0.4922 |

## Maps for the individual species distribution models.

The habitat suitability maps from the six different models for the competent mosquito species, which the consensus model is based on, are shown in S4.


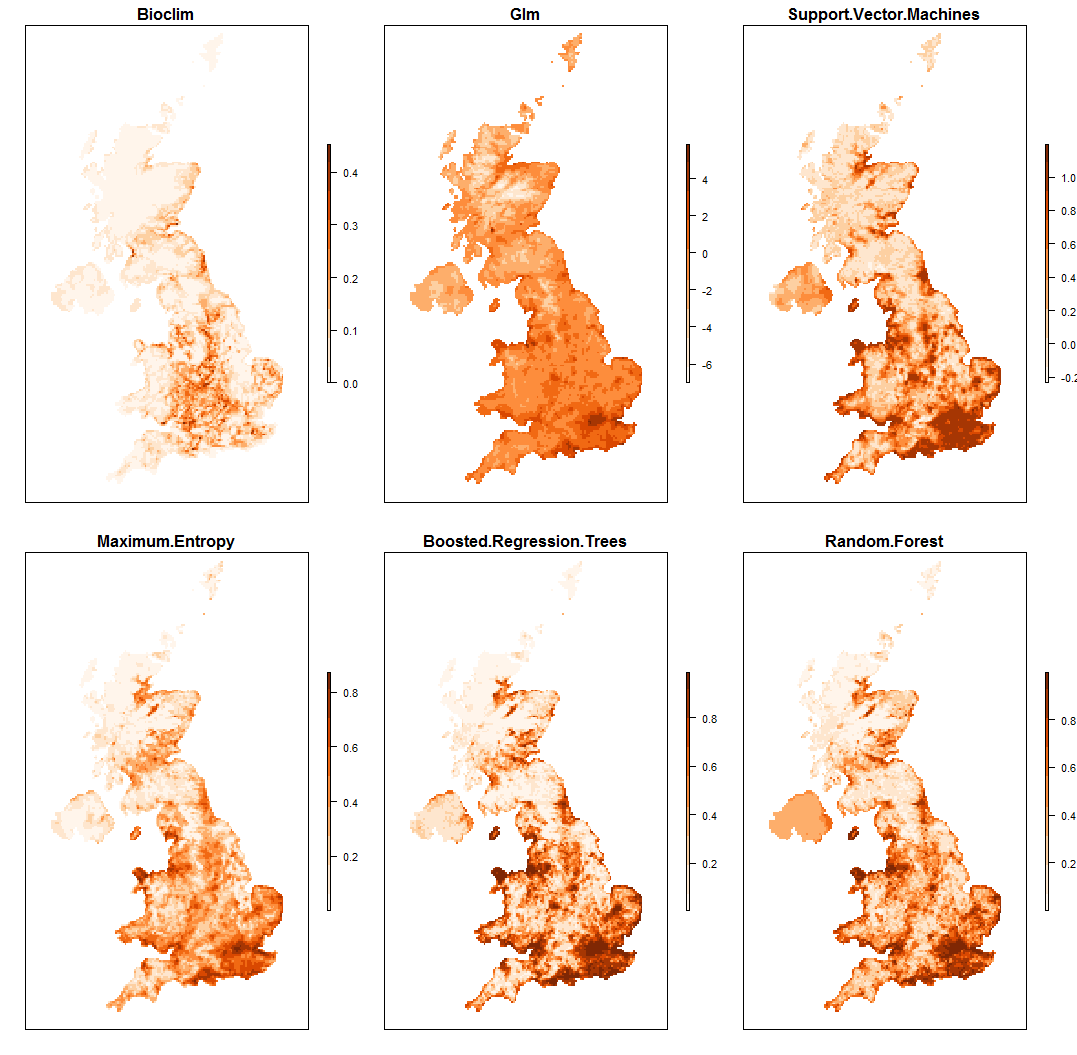


Figure S4: Maps of the mean habitat suitability scores for presence of RVFV susceptible mosquito species in UK, using six different modelling approaches. Note that scales are different so absolute values are not directly comparable between models.

Figure S5 shows the number of iterations where the areas are above the threshold values for the different models.


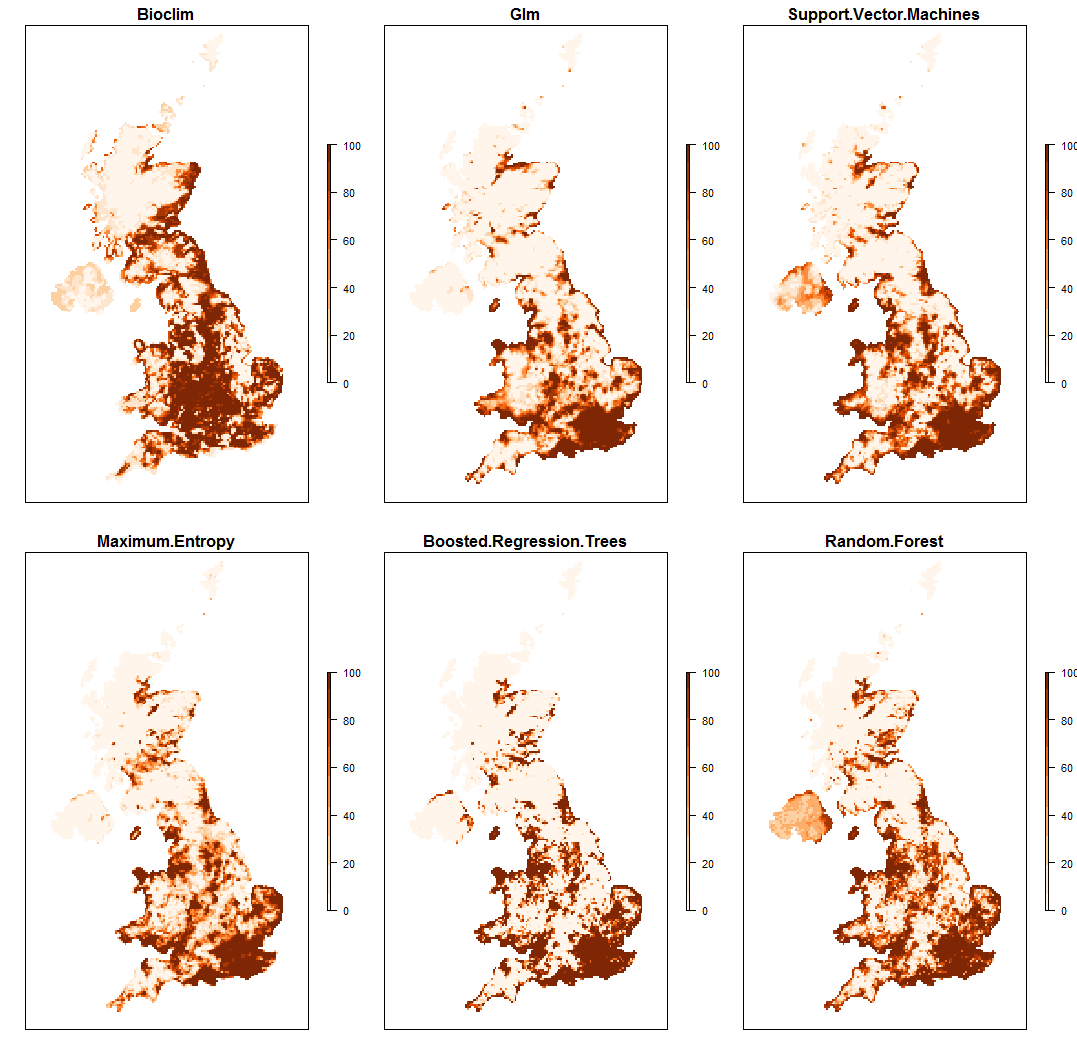


Figure S5: Areas above the threshold value for presence of RVFV susceptible mosquito species in UK, using six different modelling approaches. Results are number of iterations (out of 100) where the area is above the threshold value.

Figure S6 shows the consensus model output map with the presence and absence points used in the model. Note that absence points are defined as raster cells where Culex species have been observed, but RVF competent species have not. It is possible for RVF competent species and Culex spp. to be found at the same point.


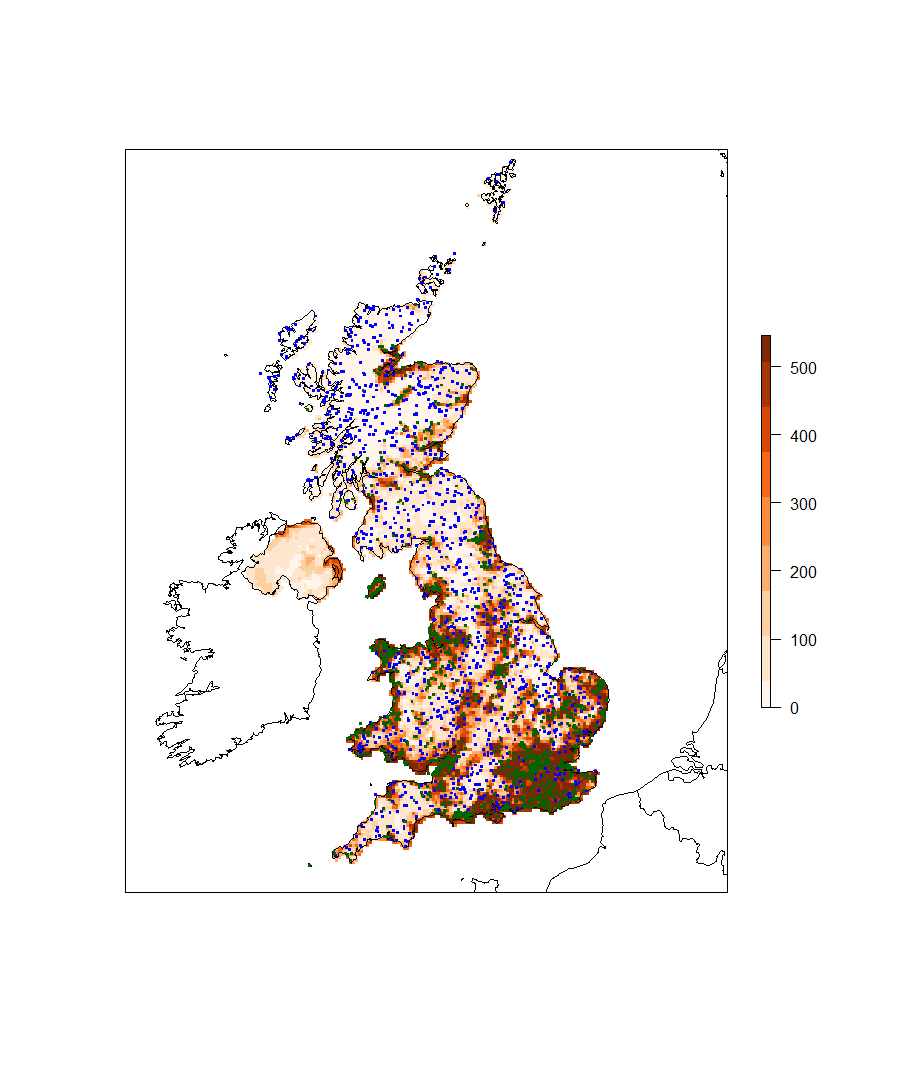


Figure S6: Consensus model map, with points identifying presence and absence cells, higher values (darker shade) indicate higher model predicted relative suitability for competent mosquito species. Cells with observed competent mosquitos are shown by the green dots, with absence cells denoted by blue dots.

## Scenario including seasonal climatic variables.

By using only yearly average estimates of data from the UK met office in the species distribution models, there is the risk of not accounting for potentially important effects due to seasonality, which could impact the accuracy of the model. To address this we ran a scenario where we added climatic variables derived from the Met Office climate data, based on those from the WorldClim dataset (WorldClim, 2019). The WorldClim dataset consisted of 19 climatic variables:

BIO1 = Annual Mean Temperature
BIO2 = Mean Diurnal Range (Mean of monthly (max temp - min temp))
BIO3 = Isothermality (BIO2/BIO7) (* 100)
BIO4 = Temperature Seasonality (standard deviation *100)
BIO5 = Max Temperature of Warmest Month
BIO6 = Min Temperature of Coldest Month
BIO7 = Temperature Annual Range (BIO5-BIO6)
BIO8 = Mean Temperature of Wettest Quarter
BIO9 = Mean Temperature of Driest Quarter
BIO10 = Mean Temperature of Warmest Quarter
BIO11 = Mean Temperature of Coldest Quarter
BIO12 = Annual Precipitation
BIO13 = Precipitation of Wettest Month
BIO14 = Precipitation of Driest Month
BIO15 = Precipitation Seasonality (Coefficient of Variation)
BIO16 = Precipitation of Wettest Quarter
BIO17 = Precipitation of Driest Quarter
BIO18 = Precipitation of Warmest Quarter
BIO19 = Precipitation of Coldest Quarter

These variables were calculated from the Met Office data and then added as new predictors to the raster stack from the baseline model. Once the full raster stack was collated, we then ran the full consensus model as per the baseline model. The vif selection process allowed for selection/rejection of both the previous predictors and the new predictors. This process only selected 6 of the 19 new variables, most of these were to do with seasonality of temperature or precipitation: Isothermality (BIO3), precipitation of both the driest and wettest months (BIO13 and BIO14), Min Temperature of Coldest month (BIO6), and temperature and precipitation seasonality (BIO4 and BIO15).

Visual inspection of the final consensus model suggested there very little difference from the baseline model (Fig S7). The goodness of fit statistics were a little bit better (the mean AUC increased to 0.887, from 0.882, and the mean model value at the presence points to 0.8496, from 0.845). This suggests, that in this case incorporation of the seasonality statistics do not noticeably improve the accuracy of the model, but this should be assessed on a case by case basis as they may be more influential in other situations, e.g. for a different pathogen.

**
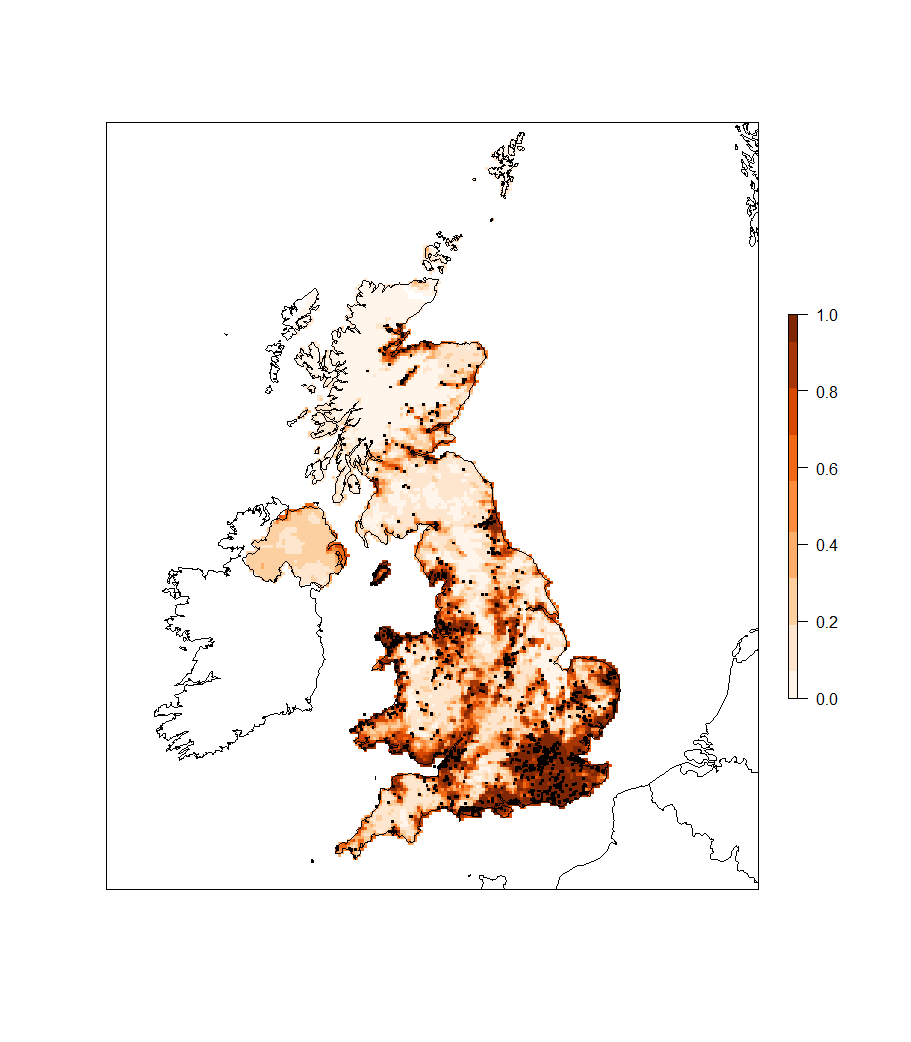
**
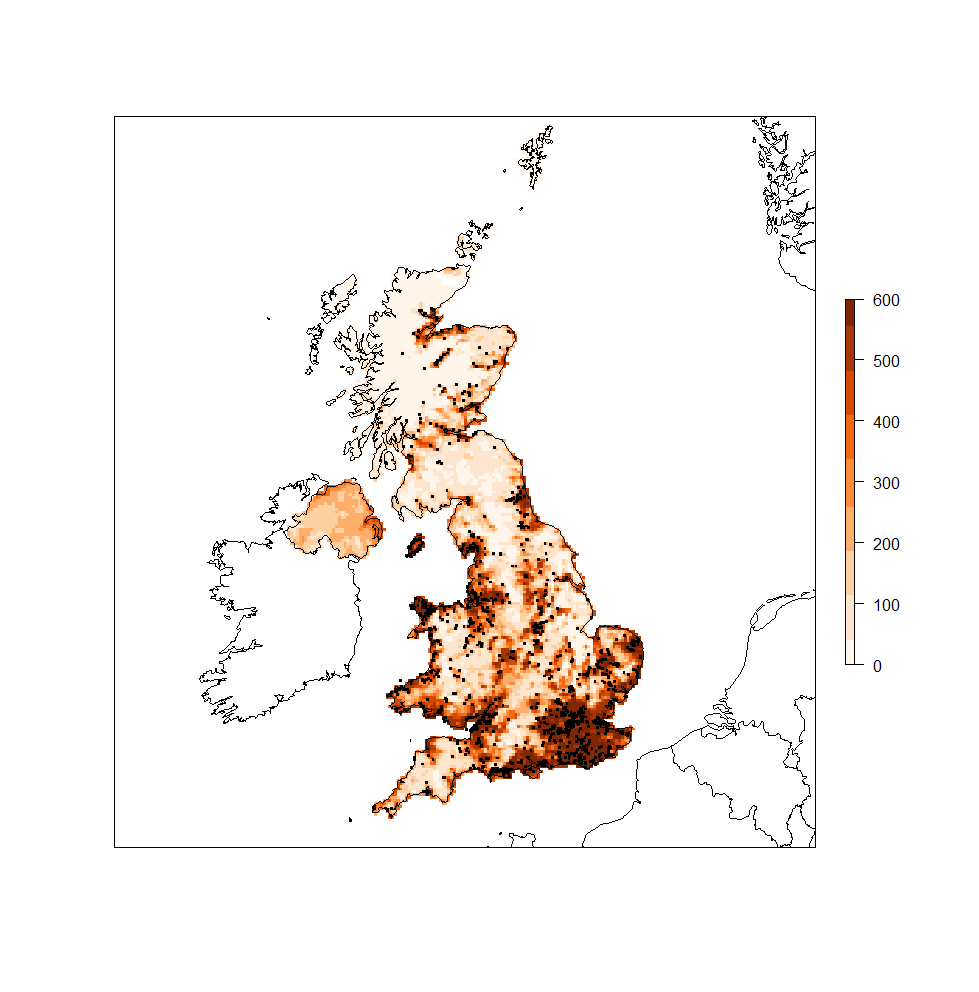


Figure S7: Baseline consensus model map (left) and consensus model including predictors derived from the Met Office based on WorldClim variables (right). Models indicate probability of presence of RVF competent mosquito species. Higher values closer to 1 (darker shade) indicate higher relative probability of presence of competent mosquito species compared to lower values. Observed mosquito presence data are shown by the black squares.

**References**

WorldClim (2019). Dataset from WorldClim, <https://www.worldclim.org/bioclim>, downloaded on 15/06/19.
